# Hemorrhagic Fever with Renal Syndrome in Asia: History, Pathogenesis, Diagnosis, Treatment, and Prevention

**DOI:** 10.3390/v15020561

**Published:** 2023-02-18

**Authors:** Ayushi Sehgal, Sanya Mehta, Kritika Sahay, Ekaterina Martynova, Albert Rizvanov, Manoj Baranwal, Sara Chandy, Svetlana Khaiboullina, Emmanuel Kabwe, Yuriy Davidyuk

**Affiliations:** 1 Department of Biotechnology, Thapar Institute of Engineering and Technology, Patiala 147004, India; 2 OpenLab “Gene and Cell Technologies”, Institute of Fundamental Medicine and Biology, Kazan Federal University, Kazan 420008, Russia; 3 Childs Trust Medical Research Foundation, Kanchi Kamakoti Childs Trust Hospital, Chennai 600034, India; 4 Kazan Research Institute of Epidemiology and Microbiology, Kazan 420012, Russia

**Keywords:** emerging viruses, orthohantaviruses, therapeutics, vaccines, immunology

## Abstract

Hemorrhagic Fever with Renal Syndrome (HFRS) is the most frequently diagnosed zoonosis in Asia. This zoonotic infection is the result of exposure to the virus-contaminated aerosols. Orthohantavirus infection may cause Hemorrhagic Fever with Renal Syndrome (HRFS), a disease that is characterized by acute kidney injury and increased vascular permeability. Several species of orthohantaviruses were identified as causing infection, where Hantaan, Puumala, and Seoul viruses are most common. Orthohantaviruses are endemic to several Asian countries, such as China, South Korea, and Japan. Along with those countries, HFRS tops the list of zoonotic infections in the Far Eastern Federal District of Russia. Recently, orthohantavirus circulation was demonstrated in small mammals in Thailand and India, where orthohantavirus was not believed to be endemic. In this review, we summarized the current data on orthohantaviruses in Asia. We gave the synopsis of the history and diversity of orthohantaviruses in Asia. We also described the clinical presentation and current understanding of the pathogenesis of orthohantavirus infection. Additionally, conventional and novel approaches for preventing and treating orthohantavirus infection are discussed.

## 1. Introduction

Orthohantaviruses, are zoonotic pathogens belonging to the genus *Orthohantavirus* family *Hantaviridae* [1,2]. Multiple members of the genus *Orthohantavirus* are human pathogens: Hantaan virus (HNTV), Seoul virus (SEOV), Puumala virus (PUUV), and Dobrava-Belgrade virus (DOBV). Presently, 38 orthohantavirus species have been identified [1], out of which at least 24 are capable of causing infectious diseases in humans [3]. Orthohantaviruses can cause two acute febrile diseases: Hemorrhagic Fever with Renal Syndrome (HFRS) and Hantavirus Cardiopulmonary Syndrome (HCPS) [4,5]. HFRS and HCPS are confined to the geographic distribution of natural small mammal hosts [4]. The group of orthohantaviruses which cause HFRS to circulate primarily in small rodents inhabiting the European and Asian regions [6,7,8].

**Figure 1:**
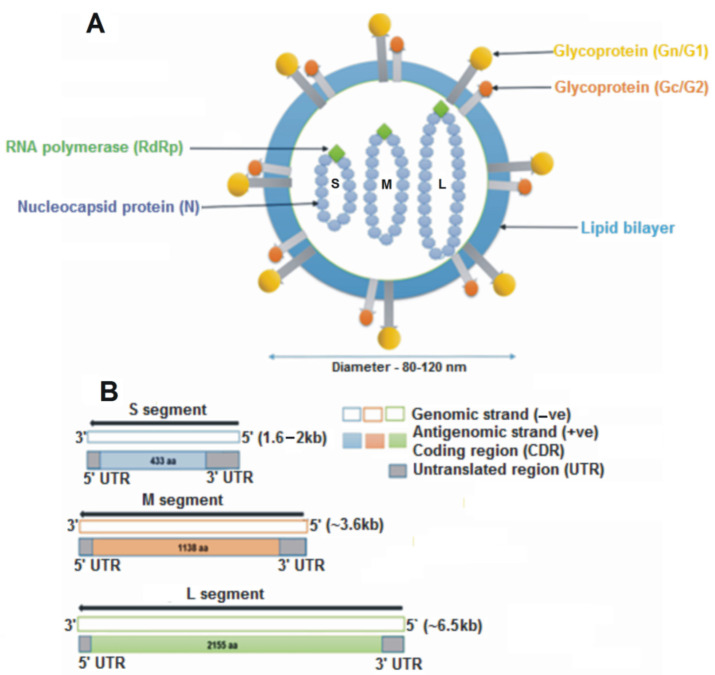
Schematic representation of an orthohantavirus virion. (**A**) The structure is spherical, having a diameter of 8–120 nm, enveloped with a lipid bilayer containing spikes of glycoproteins (Gn and Gc). Inside the virion are three segments of single-stranded RNA: small (S), medium (M) and large (L). (**B**) The S, M, and L genomic segments encode for the nucleocapsid protein (433 aa), glycoprotein precursor (1138 aa) and the RNA-dependent RNA polymerase (2155 aa), respectively.

Orthohantaviruses have a negative sense single-stranded RNA genome, which is organized into three segments: small (S), medium (M), and large (L) (Figure 1). These RNAs encode nucleocapsid (N) protein, glycoprotein precursor (GPC), which is processed into two glycoproteins (Gn and Gc), and the viral RNA-dependent RNA polymerase (RdRp) protein, respectively [9].

Orthohantaviruses are endemic in many Asian countries, with the largest number of cases reported in China, South Korea, and the Far Eastern Federal District of Russia [9]. This review provides a history of the orthohantaviruses and the disease they cause in Asia, with particular emphasis on the clinical manifestation and pathogenesis. We will also address challenges in the prevention, treatment, and diagnosis of orthohantaviruses in Asia.

## 2. History of Orthohantaviruses and the Disease They Cause in Asia

In China, febrile disease with renal dysfunction was documented as early as 1931 [4]. Since then, HFRS has been acknowledged as an endemic disease in this country, where 40–50% of worldwide HFRS cases are diagnosed [9]. China remains the most active HFRS endemic region, where cases increased from 10,378 reported in 1931–1949 [10] to 1,557,622 diagnosed between 1950 and 2007 [11]. In recent decades, the incidence of HFRS in China has decreased. During the period 2004–2019, a total of 209,209 cases of the disease were registered, of which 1855 were fatal [12]. Epidemiological analysis indicated several “hot spots” in China with a high risk of orthohantavirus infection: Shandon, Heilongjiang, Hunan, Jiangxi, and Zhejiang provinces [4].

Still, HFRS is endemic in the Far Eastern Federal District of Russia, where 3145 cases were reported in 15 of the 29 regions of Asian Russia between 1978 and 1995 [4]. The endemic orthohantavirus has a long history in this region, with the first HFRS cases reported in 1934 in Khabarovsk, Primorsky Krai, and the Amur Region. Since then, cases of HFRS have been reported in other regions of Russia [13]. It was suggested that in Asian Russia the disease was only limited to the Far East region of Russia [14,15,16,17].

Symptoms like HFRS were also described in approximately 1700 US soldiers during the Korean War (1950–1953), with less than 5% resulting in deaths [18]. Later, symptoms similar to HFRS from the Far East region were noticed in patients from Scandinavia [15]. At that time, knowledge of the infectious agent causing HFRS remained limited [19,20]. Human infection was usually associated with exposure to excreta from small rodents [21].

The identity of an infectious agent was revealed several years after, following a retrospective study of clinical cases. In 1978, Dr. Lee isolated the etiologic agent of hemorrhagic fever from the *Apodemus agrarius* lung tissue, which reacted with convalescent serum from HFRS patients [22]. The morphology of a new virus, named the *Hantaan virus*, was identified as a member of the family *Bunyaviridae* [23].

In 1980, antibodies to the causative agent of epidemic nephropathy (NE) were detected in the bank vole [24]. Later a causative agent was identified as hantavirus and named Puumala (PUUV) after the Puumala municipality in Finland. Two years later, Lee et al. discovered a variant of the *Hantaan* virus, now known as SEOV, which caused severe HFRS in Seoul city residents [8]. Since patients had no history of traveling outside the city, urban rats were suggested as the natural reservoir of this virus in South Korea. The retrospective research showed that 15% of the rats in Seoul and 28% in Tokyo carried the newly identified virus [25].

The first cases of suspected orthohantavirus infection in India were reported in 1964 in Vellore [26]. Investigation of these outbreaks led to identifying a novel member of the genus *Orthohantavirus* named Thottapalayam virus (TPMV) [26,27]. Initially, this virus was isolated from a non-rodent host, the house musk shrew (*Suncus murinus*) [26]. Although TPMV had morphological and genetic similarities with members of the genus *Orthohantavirus* of the family *Hantaviridae*, later, TPMV was shown to form a phylogenetically distinct genus [1]. Therefore, it was assigned a new name, a *Thottapalayam thottimvirus*, which is a proto-type shrew-borne orthohantavirus that belongs to the genus *Thottimvirus*. In addition to Vellore, a high rate of orthohantavirus seropositivity was reported in healthy individuals from the Cochin and Chennai regions of India [28]. Analysis of serum samples revealed the cross-reactivity with SEOV in 12% and PUUV in 5% of individuals, respectively [28]. In another study of 152 serum samples, 23 reacted with HTNV, PUUV, or SEOV [29]. These results provide strong evidence that orthohantaviruses are endemic in India.

Hantavirus-reactive antibodies were found in serum samples from 5461 small mammals belonging to 16 different species in Taiwan [30]. It was demonstrated that *Rattus norvegicus* was the most common rodent species captured in that region, and these rodents were major contributors to orthohantavirus circulation in small mammals in the region. Using reverse transcriptase polymerase chain reaction (RT-PCR), SEOV was detected in Rattus norvegicus in lung tissues. As a result of further analyses, it was discovered that almost all orthohantavirus infections in Taiwan were caused by SEOV [30].

Also, 3.8% of rodents captured in the Nakhon Pathom region were seropositive for orthohantavirus antigens [31]. All the sera-positive rodents were *Bandicoot indica* species. It was shown that these rodents were carrying the Hantaan-like virus [31]. Antibodies to the Hantaan-like virus were found in humans living near locations of infected rodents. The positive titer of antibodies to Hantavirus varied from 5–7% to 31–33 [31]. Later this virus was named *Thailand orthohantavirus* (THAIV).

For the first time after the detection of hantaviruses, the prevailing opinion was that their carriers were rodents; however, later studies have identified hantavirus antigen (virus) or/and specific antibodies in domestic animals, such as cats, rabbits, and dogs [32]. A case of human infection with hantavirus from a domestic rat has also been reported [33]. The geographic distribution of orthohantaviruses endemic to Asia and their small mammal hosts are summarized in Table 1.

## 3. Clinical Presentation of Orthohantavirus Disease in Asia

More than 38 orthohantavirus species are known to cause human disease worldwide. While 20 are associated with the disease in Asia, the spectrum ranges from acute febrile illness with or without renal impairment, fever with shock, and multi-organ failure to hemorrhagic illness such as HFRS. Hemorrhage and acute renal injury (AKI) are common clinical manifestations in severe HFRS cases [51].

Clinical presentation of HFRS may range from subclinical, mild, and moderate to severe. HTNV, DOBV, and AMRV are more often cause severe HFRS, while the moderate form is frequently diagnosed in SEOV causes. Also, *Nephropathia epidemica* (NE), a mild form of HFRS linked to PUUV infection, is diagnosed in Russia [52].

The incubation period of HFRS generally ranges between two and four weeks; however, in some cases, it could be as long as six weeks [52]. Clinically, HFRS progresses through five phases: febrile, hypotensive, oliguric, polyuric, and convalescent. These phases are demarcated in severe HFRS but may overlap or be absent in mild and moderate forms of the disease [52,53].

The febrile phase usually lasts three to seven days and is characterized by high fever, chills, headache, backache, abdominal pains, nausea, and vomiting. Symptoms are non-specific, making orthohantavirus infection diagnosis challenging during this phase. By day three to four post-onset, hemorrhagic manifestations appear in the forms of diffuse petechiae on the conjunctiva and the palate. AKI symptoms of hematuria and proteinuria become evident by day seven of the disease [54].

Shock is typical for the severe form of HFRS. Approximately 11–40% of febrile patients develop hypotension, and one-third have a shock [52]. The hypotensive phase could last from several hours to a couple of days. Thrombocytopenia and leukocytosis are commonly documented during this phase. Also, symptoms of an AKI, such as acute tubulointerstitial nephritis, necrotizing glomerulonephritis, and IgA nephropathy, are characteristic of this phase.

The oliguric phase lasts three to seven days. Patients are at risk of hypotension, severe pulmonary edema, and AKI, as symptoms of oliguria, anuria, proteinuria, hematuria, and azotemia are commonly described. The severe form of HFRS could require hemodialysis at this phase. This is the critical phase, as half of the total fatalities occur during this phase. Typical laboratory findings are elevated serum creatinine and urea [9,55]. The polyuric phase, which could last for days or weeks, is characterized by increased urinary output. The renal function restores, and symptoms of AKI dissolve.

Convalescence is usually extended and could last up to six months. Recovery is complete, though sequelae of chronic renal failure and hypertension have been reported [56,57]. The fatality rate of 5–15% in HTNV/DOBV-related HFRS is likely due to complications, such as renal insufficiency, edema, hemorrhages, encephalopathy, and shock. SEO infection causes a moderate form of HFRS with a clinical presentation similar to HFRS caused by HTNV [58]. The SEOV infection fatality rate is 1% [59]. Even lower than 1.0% is the mortality rate of PUUV infection [60].

## 4. Pathogenesis of Orthohantavirus Infections

It is believed that orthohantavirus infection results from inhaling virus-contaminated aerosol or other forms of contact with the virus [61,62]. The initial site of orthohantavirus replication appears to be the respiratory tract. The susceptibility of human epithelial cells derived from bronchi, bronchioles, and alveoli to PUUV infection was recently in vitro [63]. The most intriguing finding was the substantial donor-specific variation in the efficacy of virus replication in respiratory epithelial cells. These data provide the basis for an individual-specific susceptibility to orthohantavirus infection.

**Figure 2:**
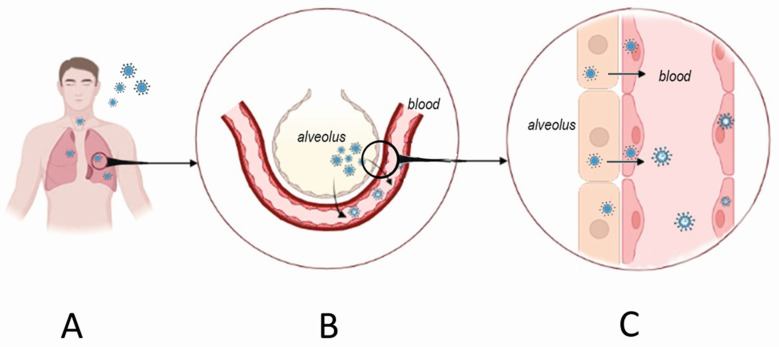
Orthohantavirus infection portal of entry and initial replication sites. Orthohantaviruses use the respiratory tract as one of the portals of entry (**A**). The initial replication site is believed to be in alveolar pneumocytes. From there, the virus crosses the respiratory membrane infecting nearby endothelial cells of the alveolar capillaries (**B**). These steps occur before the patient presents with HFRS symptoms. Once orthohantavirus infects endothelial cells, it becomes released into the blood leading to viremia (**C**). The immune system’s reaction to viremia could cause tissue damage, while increased endothelial permeability and blood coagulation could result from infected endothelial cell activation.

Infection of respiratory epithelial cells and dissemination of orthohantavirus happens early before clinical symptoms can be identified (Figure 2). Several studies have demonstrated orthohantavirus RNA in the serum of patients up to three weeks before the onset of the disease [64,65,66]. These data indicate that the virus replicates in the respiratory tissue; however, the body’s reaction to infection is delayed. The non-cytopathic nature of orthohantavirus replication could explain this delay in clinical symptoms. The lack of infected cell death was demonstrated in vitro [67,68]. Also, virus replication-explained damage was not found in tissues collected from orthohantavirus-infected patients [69,70,71]. In contrast, HNTV RNA was detected in the plasma of patients at an early stage of HFRS and the high viral load led to the correlation with the severity of the disease [72]. Also, DOBV RNA level in HFRS patients’ serum correlated with disease severity [73].

Slow virus replication capacity could contribute to delayed symptom development after the beginning of viremia [74].

Infection of endothelial cells is the next crucial step in orthohantavirus pathogenesis. Orthohantavirus most likely reaches the endothelial cells of the respiratory tract at the gas exchange membrane site first. Then, viremia follows, making endothelial cells of the small vessels the main target of infection [75]. Endothelial cells are commonly found positive for orthohantavirus antigens in biopsies, and postmortem collected tissues [63,75,76]. Endothelial cells were also susceptible to orthohantavirus infection and support virus replication in vitro [77]. For cell entry, orthohantaviruses use integrin receptors [77], which are expressed in endothelial cells [78].

Infection and replication in endothelial cells appear essential for the pathogenesis of orthohantavirus disease. There are three main consequences of orthohantavirus infection of endothelial cells: (a) loss of the endothelium barrier integrity, (b) activation of coagulation, and (c) release of cytokines and activation of the immune response. The loss of the blood tissue barrier function will increase vascular permeability leading to edema and hemorrhages [53]. Orthohantavirus infection activates endothelial cells to initiate thrombocyte aggregation and blood coagulation. Also, infection of endothelial cells could initiate the release of various cytokines with the potential to induce inflammation, activate the immune response, and sustain the impaired permeability of the endothelium.

**Figure 3:**
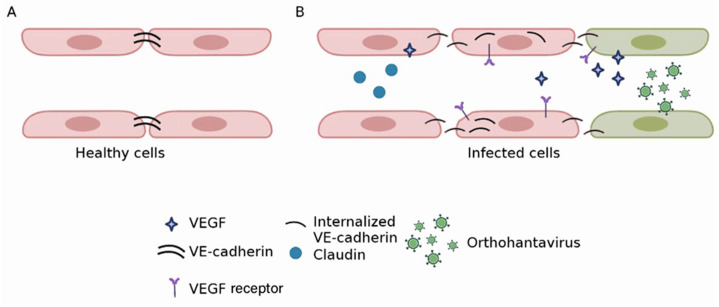
Orthohantavirus effect on endothelium barrier integrity. In uninfected endothelial cells, VE-cadherin is expressed in junctions between adjacent cells (**A**). In orthohantavirus-infected cells, a profound reorganization of VE-cadherin was described (**B**). It was shown that VE-cadherin becomes internalized in orthohantavirus-infected endothelial cells. This change in molecule expression appears to be a response to the binding of infected cells to VEGF. As a result, the integrity of adherence junctions becomes compromised, leading to increased endothelium permeability. Expression of claudin, a TJ molecule, decreased in orthohantavirus-infected cells. This would also contribute to the disintegration of cell adhesion and vascular leakage.

*Loss of endothelium barrier function*: Orthohantaviruses endemic in Asia use beta integrin receptors to adhere to and penetrate endothelial cells [79]. Virus replication is demonstrated in vitro and in vivo without cytopathic effect [67,68,80]. Therefore, the loss of the barrier function is not essential for virus replication but rather the result of the endothelial cells’ response to infection (Figure 3). This assumption is supported by an in vitro study by Gorbunova et al., which demonstrated vascular endothelial (VE)-cadherin internalization in HNTV-infected endothelial cells [81]. In another study, Wang et al. demonstrated the formation of a functional complex between β3 integrin and VEGFR2 in HNTV-infected cells [82]. This complex could initiate signaling leading to cytoskeleton reorganization and subsequently, hyperpermeability. These authors also confirmed the role of VEGF in the disruption of junctions between endothelial cells. Decreased expression of Claudin-1, a tight junction (TJ) component [83], was found in HNTV-infected endothelial cells [84]. Another molecule part of the TJ zona occludens 1 (ZO-1) was altered in HFRS kidney biopsies [85]. Krautkramer et al. reported decreased expression of ZO-1 in the kidney tubular epithelium of orthohantavirus-infected patients [85]. Recently, the role of protocadherin one as an orthohantavirus receptor was demonstrated by Dieterle et al. [86] and Jangra et al. [87]. Its involvement in virus entry supports the hypothesis of the profoundly disturbing endothelial monolayer integrity in orthohantavirus-infected patients. Together, these data provide strong evidence for the substantial destruction of the structures maintaining endothelium integrity.

Data in vivo supports increased endothelial permeability in orthohantavirus-infected patients. These include finding a decreased serum level of the glycocalyx in HFRS [88]. Also, a syndecan-1 level was found to decrease HFRS. Additionally, a lower serum level of pro-angiogenic angiopoietin 1 was demonstrated in HFRS [89].

*Activation of coagulation*: Decreased expression of von Willebrand (VW) factor in HNTV-infected endothelial cells was demonstrated by Cho et al. [90]. Also, we demonstrated the inhibition of thrombospondin 1 in HNTV-infected endothelial cells in vitro [91]. Our data corroborate Lain et al. findings of decreased thrombospondin 1 level in PUUV-infected patients [61]. Thrombospondin 1, secreted by endothelial cells [92], can directly affect fibrin degradation [93] and cleave vWF [94]. In the absence of thrombospondin 1, ultra-large vWF complexes could cause a spontaneous aggregation of platelets and thrombosis [94].

There is evidence of disturbed hemostasis in orthohantavirus-infected patients. Thrombocytopenia is an early and most consistent sign of HFRS, indicating disturbed hemostasis [95,96]. It appears that thrombocytopenia contributes to the pathogenesis of HFRS. Nadir thrombocyte counts were explained by platelet consumption and decreased survival time [97,98]. Wang et al. found a correlation between low thrombocyte counts and AKI [57]. A similar conclusion was made by Rasche, et al., in 15 PUUV convalescent patients [99]. In contrast, only a correlation between thrombocytopenia, the severity of inflammation, and capillary leakage was found by Outinen et al. [95], while the severity of AKI was independent of nadir thrombocyte counts.

Coagulation is activated in the acute phase of the infection [88,100]. An increased level of circulating prothrombin fragments 1 + 2, the fibrin D-dimers as well as consumption of the anticoagulant antithrombin was also demonstrated in acute orthohantavirus-infected patients [97,101,102]. An elevated serum level of these factors could indicate a risk of thrombosis [101]. This assumption is supported by Connolly-Andersen et al., who demonstrated a high risk of thromboembolism in post-HFRS patients [103]. Analysis of coagulopathy in HFRS demonstrated prolonged prothrombin time and activated partial thromboplastin [98]. Also, decreased levels of coagulation factors II, V, VIII, IX, and X and an increased serum level of fibrinogen were shown to correlate negatively with thrombocytopenia [61,100].

**Figure 4:**
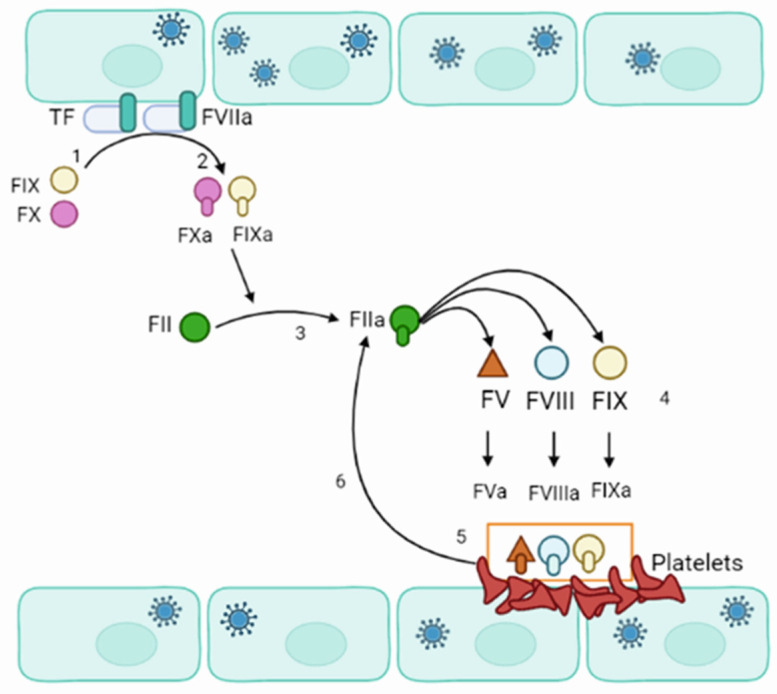
Schematics of orthohantavirus effect on blood coagulation. (1) Orthohantavirus-infected endothelial cell express TF. (2) TF interacts with active FVII (FVIIa) and calcium to convert FIX and FX to active IXa and Xa, respectively. FXa binds to factor II to form the thrombin (FIIa). (3) Thrombin interacts with FV, FVIII, and FXI. (4) Thrombin activates FV, FVIII, and FXI, forming FVa, FVIIIa, and FXIa. (5,6) FVIIIa forms a complex with FVa and FXa, which acts as a prothrombinase and accelerates the formation of thrombin (FIIa).

Blood coagulation is a process where dynamic and complex interactions between platelets and endothelial cells lead to the formation of the initial platelet plug [104,105] (Figure 4). Typically, the vascular endothelium has anti-thrombotic properties due to the expression of heparin-like glycosaminoglycans, secretion of platelet inhibitors, coagulation inhibitors, and fibrinolysis activators [106]. However, when endothelial cells are injured or activated, they can express the Tissue Factor (TF) [107,108]. TF initiates the extrinsic pathways of blood coagulation. This pathway appears to be commenced in orthohantavirus-infected patients as increased expression of TF was demonstrated in orthohantavirus-infected endothelial cells [109].

Additionally, an increased activity level of the circulating extracellular vesicle tissue factor was shown in HFRS [110]. More pro-coagulation of endothelial cells in orthohantavirus-infected patients is supported by finding glycocalyx degradation in HFRS [88]. Exposed TF could bind to factor VIIa and calcium to support the conversion of factors IX and X to active IXa and Xa, respectively [111]. Factor Xa binds to factor II to form the thrombin (factor IIa) [112]. Thrombin signals platelet activation and aggregation. Thrombin activates factors V, VIII, and XI on the platelet’s surface. The active VIIa forms a complex with active Va and Xa, which acts as a prothrombinase and accelerates the formation of Xa and thrombin. That will generate a large amount of thrombin and cleaves fibrinogen to fibrin monomers. Fibrin monomers start polymerizing and forming fibrin, the coagulation cascade’s final product.

**Figure 5:**
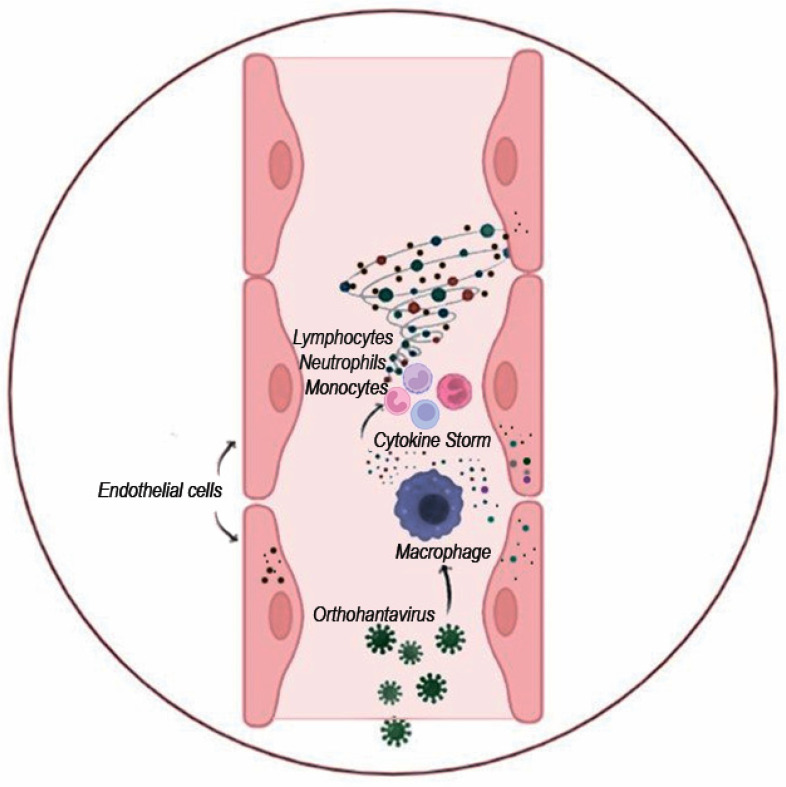
Schematics of “cytokine storm” activation upon orthohantavirus infection. Orthohantavirus infection activates endothelial cells and leukocytes to produce various cytokines. These cytokines could activate leukocytes and attract them to the site of infection. Massive infiltration of leukocytes producing proinflammatory cytokines and chemoattractants could induce severe local inflammation inflicting tissue destruction.

*Release of cytokines and activation of the immune response*: Human endothelial cells are mainly targeted by orthohantaviruses. Infections of these cells appear to be essential for compromised endothelium barrier function. Endothelial cells were also shown to produce and release cytokines, which was demonstrated in vitro [113,114]. After the initial replication in the lung epithelium, orthohantaviruses could infect the alveolar macrophages in proximity [115] (Figure 5). In addition, they could infect other cells, especially those initiating and propagating the immune response: macrophages and dendritic cells [116,117]. These leukocytes could disseminate the virus to other sites, deliver virus antigens to the lymph nodes, and contribute to the systemic cytokine release and “cytokine storm”. It should be noted that the systemic release of cytokines could further affect the endothelial barrier permeability and facilitate leukocyte adhesion and extravasation [118].

Early activation of cytokines in HFRS was evident, as their high serum level is commonly detected [119,120,121]. An increased serum level of IL-1β, IL-6, and TNF-α [122], pleiotropic cytokines was demonstrated in patients [123]. Each of these cytokines contributes to the pathophysiology of inflammation, while together, they can potentiate each other’s effect. This synergistic effect of IL-1β, IL-6, and TNF-α is evident in their pyrogenic effect. All three are endogenous pyrogens [124], where IL-6 is required for TNF-α-induced fever [125]. Overall, IL-1β and TNF-α have multiple overlapping functions. TNF-α and IL-1β could increase endothelium permeability [115,126,127]. These combined effects of TNF-α and IL-1β could establish and support the leaky endothelium [126]. IL-1β produces rapid upregulation of TF expression [126], which could contribute to coagulopathy in orthohantavirus-infected patients.

Interestingly, TF expression is also supported by IL-6 [128], indicating a synergistic effect with TNF-α and IL-1β. IL-6 also contributes to inflammation by rapid induction of acute phase proteins produced in the liver [129]. Increased serum levels of C-reactive protein (CRP) [130] and fibrinogen [131], acute phase response proteins, are commonly found in orthohantavirus-infected patients.

In addition to the activation of inflammation, each cytokine has a distinct function. IL-1β is a product of activated inflammasome [132] contributing to developing Th1 and Th17 immune responses. IL-1β supports T cells priming [133] to release IFN-γ. This combination of IL-1β and IFN-γ promotes Th1 immune response [134]. In contrast, when IL-1β is combined with IL-6, the immune response is skewed towards the Th17 type [135,136]. IL-6 also has specific functions, such as stimulation of B cell proliferation [137] and thrombopoiesis [138]. Together, increased serum level of these cytokines suggests activation of Th1 and Th2 type immune responses and thrombocyte production. Clinical laboratory findings support these conclusions: detection of circulating CD8+ lymphocytes [81], increased serum level of anti-orthohantavirus antibodies [139], and activated thrombopoiesis [140].

Chemokines attract leukocytes to the site of infection and can direct the recruitment of specific populations, thus influencing the development of the specific immune response. Activation of the subset of cytokines functionally identified as chemokines is demonstrated in HFRS and NE [122,141,142]. These chemokines, such as CCL4, CCL5, CXCL9, CXCL10, and CXCL12, regulate leukocyte recruitment across the endothelium into the tissue [143]. Chemokines commonly found activated in HFRS and NE attract mononuclear cells (CCL2 and CCL5) with a preference for Th1 lymphocytes, natural killer (NK) cells, and CD8+ lymphocytes (CXCL9 and CXCL10) [144,145,146]. These Th1 lymphocytes and NK cells are essential for protection against virus infection [147,148], especially against orthohantaviruses, which are non-cytopathic [149]. Studies have shown that CD8+ cells from convalescent HFRS could identify and eliminate orthohantavirus-infected endothelial cells [150,151]. The role of these cells in the pathogenesis of the disease is supported by the demonstration of the association between CD8+ lymphocyte count and disease severity [152]. Additionally, the potential contribution of these cells to the pathogenesis of kidney damage was shown by Temonen, et al. [153]. However, the role of CD8+ T lymphocytes in the pathogenesis of the severe form of HFRS could be more complicated, as Wang et al. has demonstrated a higher frequency of IFN-γ producing T cells in patients with a mild and moderate form of HFRS [154]. This data implies that orthohantavirus-specific CD8+ lymphocytes could play a protective role, as was also shown by Tang et al. [155]. In another study, the decline in circulating CD8+ lymphocyte counts correlated with lower virus titer, suggesting their protective role in the pathogenesis of orthohantavirus infection [156].

## 5. Vaccines and Prevention of Hantavirus Infections

Treatment of orthohantavirus infection is supportive and not specific [62,157]. Therefore, the main form of orthohantavirus control is to prevent infection. Prevention measures include minimizing exposure to rodents and utilizing vaccination.

*Minimizing human exposure*: Inhaling virus-contaminated aerosol or direct contact with rodent excreta are routes of orthohantavirus infection [158]. In order to minimize exposure, measures should be taken to prevent small rodent entry into buildings. [55,159,160]. Also, rodent control inside and around the home and workplaces will reduce contact with infected small mammals. Rodent urine and droppings should be removed regularly [161]. Clean-up starts with ventilation of the space for at least 30 min [52], followed by spraying and soaking urine and droppings with disinfectant before removal.

Monitoring the rodent population by local authorities is essential for predicting and preventing orthohantavirus outbreaks. There are multiple small mammals identified in Asia that could carry HNTV, SEOV, and THAIV [162]. These carrier mammals include *Apodemus agrarius*, the primary reservoir host for HTNV [163], and *Rattus norvegicus*, the carrier for SEOV [164]. Also, *Bandicota indica*, *Rattus rattus*, and *Eliurus major* rat species should be included in the monitoring as they are shown to be reservoirs for THAIV [31,165,166]. Studies have shown that the density of the population could affect the horizontal transmission of orthohantavirus, increasing the number of infected small rodents [167,168]. It was also demonstrated that the threshold density of the rodent population is required to maintain orthohantavirus [169,170,171]. A higher number of infected small mammals could potentially increase the chance of human contact and exposure to orthohantavirus. Therefore, the efficacy of zoonotic carrier population control remains a necessary measure to prevent orthohantavirus infection.

*Hantavirus vaccine*: There is no World Health Organization (WHO) approved vaccine to prevent orthohantavirus infection. Likewise, local authorities do not approve the orthohantavirus vaccine in endemic areas such as Europe and North and South America [172]. However, inactivated orthohantavirus vaccines are licensed in China and Korea [173]. Inactivated rodent brain or cell culture-derived HFRS vaccines are used in China [11,174]. In 1993, the first inactivated orthohantavirus vaccine was approved in China [11]. Since then, four inactivated HTNV, and SEOV-based vaccines have been used in China and demonstrated the safety and efficacy of protection [175]. Started in 1990, an inactivated HFRS vaccine has also been used in Korea [176]. Since the beginning of vaccination, the number of HFRS cases has reduced significantly [176]. The vaccine’s effectiveness was also reported by Park et al.; however, the authors state that using a large cohort and a long monitoring period is required to make conclusions regarding vaccine efficacy [177].

During the 30 years of the HFRS vaccine development, novel approaches were developed to improve the delivery, orthohantavirus antigens expression, the efficacy of the immune system activation, and reducing side effects.

*Inactivated vaccines*: These were the first type of vaccines containing inactivated orthohantavirus virion [176]. HNTV was propagated in the brains of suckling mice, followed by chemical inactivation before being tested for immunogenicity [178]. In 1990, this vaccine, under the commercial name Hantavax, was tested in a clinical trial where safety and seroconversion of over 90% was demonstrated [179,180]. Neutralizing antibodies were also demonstrated in 75% of vaccinated one month after the booster [179]. Lesser antibody prevalence, 23 and 41%, as demonstrated in several studies [180,181]. Studies have demonstrated anti-orthohantavirus antibodies several months after immunization [181,182]. Also, the immune response was detected one year after immunization [180]. The protective efficacy of the Hantavax vaccine was suggested to explain the decline in the number of HFRS cases in South Korea between 1991 and 1997 [176]. Later studies aimed to analyze the effect of the Hantavax vaccine to affect the disease progression and demonstrated the reduction of stage 3 acute kidney injury and the requirement for dialysis in the vaccinated cohort [183]. In another study, the efficacy of the anti-orthohantavirus vaccine was demonstrated in an immunized cohort from Yugoslavia [184]. Also, the developers of Hantavax, in collaboration with the Yugoslavia research team, demonstrated the vaccine’s protective efficacy [177].

Inactivated hantavirus vaccines in South Korea demonstrated controversy regarding their effectiveness [185]. In contrast, studies of vaccines in China showed that the absorbance value of HFRS-IgG was four times higher in vaccinated persons than those nonimmunized in the epidemic areas [186]. Also, later, the protective effectiveness of the three-doses regimen of the inactivated HFRS vaccine compared to the two-doses regimen was demonstrated [187].

Bivalent, HNTV, and SEOV inactivated orthohantavirus vaccines were manufactured in China [188]. The persistence of anti-orthohantavirus antibodies was demonstrated after a three-dose series in China [187]. In another study, the bivalent inactivated vaccine induced anti-orthohantavirus antibodies one month after immunization [189]. Antibodies were detectable 33 months after vaccination. Recently, pre-clinical studies of inactivated polyvalent HFRS vaccine demonstrated the activation of a balanced immune response to PUUV, HNTV, and DOBV [190].

*Virus-like particle (VLP) vaccine*: Inactivated vaccines effectively activate the humoral immune response. However, these vaccines had substantial limitations, such as failure to induce long-term antibody response, multiple immunizations, and potential side effects. Therefore, there was still an interest in developing vaccines that could address these obstacles. VLP vaccine could provide a solution to some of these limitations. Several VLP is nano-sized self-assembly competent structures made by viral proteins [191]. They have spike proteins, which could bind to the host receptor [192,193]. Therefore, VLP host cell entry resembles a natural infection. However, VLPs lack nucleic acid, rendering them incapable of replication [191]. As a result, VLPs could deliver the viral antigens to the host cells without virus replication and, subsequently, disease symptoms. This feature of VLP made them an attractive tool for developing second-generation vaccines.

The efficacy of the VLP containing HNTV N and Gn/Gc proteins was tested in a mouse model [194]. To enhance the immunogenicity, HNTV chimeric VLPs containing glycosylphosphatidylinositol (GPI)-anchored granulocyte-macrophage colony-stimulating factor (GM-CSF) or CD40 ligand (CD40L) were generated. These chimeric VLPs induce humoral and cellular immune responses, which are more potent than HNTV VLP or commercially available inactivated vaccines. It should be noted that chimeric VLPs also protected mice from the HTNV challenge. Cheng et al. obtained similar results using GM-CSF-CD40L chimeric VLP [194]. Additionally, authors have demonstrated that incorporating GM-CSF-CD40L stimulated macrophages and dendritic cells. In another study by Dong et al., chimeric VLPs were shown to induce long-term immune responses with neutralizing antibodies circulating six months after immunization [195].

Production of anti-orthohantavirus antibodies was evidenced after immunization with chimeric hepatitis B virus (HBV) particles containing PUUV N protein polypeptide [196]. These chimeric HBVs expressing PUUV N protein polypeptide induced a protective immune response in bank voles, the natural reservoir of the PUUV [197]. In another study, the immunogenic efficacy of HBV core particles carrying the N protein polypeptide of the DOBV, HNTV, or PUUV in a mice model was demonstrated [198]. These chimeric particles induced a high titer of cross-reactive antibodies [199].

*DNA vaccine*: DNA vaccines have multiple advantages as compared to inactivated vaccines. DNA vaccines are safe, as they are replication defective. They are also non-virulent and fail to produce clinical symptoms of the disease. As early as 1992, the HNTV DNA vaccine expressing N and G proteins was developed using the vaccinia virus as a vector [200]. This vaccine elicited a protective immune response in a hamster model. The same vaccine was later proved protective against HNTV, SEOV, and PUUV [201].

Interestingly, anti-HNTV neutralizing antibodies were detected in immunized animals. In contrast, there were no antibodies to the SEOV virus. In clinical trials phase I, neutralizing antibodies was demonstrated in immunized individuals. However, previous exposure to the vaccinia virus appears to interfere with the efficacy of developing neutralizing antibodies in volunteers [198].

DNA vaccines appear to be immunogenic and induce neutralizing antibody responses. For example, two DNA vaccines, HTNV and PUUV, were tested in phase I clinical trials [202]. Both vaccines elicited neutralizing antibodies; however, only about half vaccinated were seropositive. DNA vaccine against SEOV induced antibody response in Syrian hamsters and protected against infection [203]. Rhesus monkey immunization with another DNA vaccine, coding for HNTV and ANDV M genes, was shown to induce neutralizing antibodies [204]. Neutralizing antibodies can bind to envelop proteins and prevent viral entry [205,206]. This reduces virus infectivity and prevents dissemination. The ability to elicit neutralizing antibodies is an established benchmark for assessing vaccine efficacy.

*Subunit vaccines*: Orthohantavirus Gn/Gc and N proteins could elicit a strong humoral immune response [207,208,209]. Gn/Gc proteins could induce neutralizing antibody response [210], while N protein activates non-neutralizing antibody [211]. Additionally, the N protein stimulates the T cell immune response [212,213]. N protein was shown to induce the cross-reacting immune response [150,214]. Similarly, recombinant DOBV N protein was shown to induce antibodies cross-reacting with PUUV and HNTV in mice [215]. In another study, immunization with SEOV recombinant N protein induced high-titer antibody [216]. Recently, the efficacy of eliciting humoral and T cell immune response by delivery of PUUV Gn/Gc and N protein using microvesicles was demonstrated [217]. However, the efficacy of these recombinant proteins’ protection against lethal infection remains to be determined [218].

## 6. Treatment of Orthohantavirus Infection

There are no specific post-exposure therapeutics for HFRS. Therefore, treatment may differ between healthcare facilities adapting to patient management protocols based on local regulatory authority’s recommendations. Therapeutics to treat HFRS could be classified as targeting viruses, immune response, and supportive therapy.

Therapeutics targeting viruses could block virus entry and replication. Neutralizing antibodies can bind to surface glycoproteins, preventing binding to integrin receptors. Transfer of orthohantavirus convalescent serum containing neutralizing antibodies was shown to have a protective effect in the non-randomized multi-center trial [219]. Also, the protection efficacy of convalescent serum was confirmed in vivo experiments [220,221]. Several monoclonal antibodies with neutralizing activity were generated against HNTV [203,221]. Phase I and II clinical studies have demonstrated the therapeutic efficacy of these neutralizing antibodies in the early stage of HFRS [221,222].

Novel therapeutic approaches targeting orthohantavirus entry are still in the development stage. One is based on the peptides binding to αvβ3 integrin receptors [223]. Orthohantaviruses bind to this integrin receptor for cell entry [79]. Therefore, the initial interaction between orthohantavirus and the integrin receptor is crucial in the virus replication process. By preventing this interaction, virus entry could be abrogated, protecting cells from infection. A study by Song et al. supported this hypothesis, where monoclonal antibodies to β3 integrin protected mice from HNTV infection [224]. This concept was further developed by Hall et al., where peptides binding to integrin receptors were shown to block orthohantavirus entry [225]. A follow-up study demonstrated the efficacy of neutralizing SNV, ANDV, and HTNV by selected cyclic nonapeptides [223]. In vivo studies could evaluate the therapeutic efficacy of these peptides against orthohantaviruses.

Another approach targets virus replication. One of the earliest drugs tested for its therapeutic efficacy against the orthohantavirus infection is Ribavirin. Ribavirin is a nucleotide analog (1-beta-D-ribofuranosyl1,2,4-triazole-3-carboxamide) used to treat HFRS [226,227]. The primary mechanism of antiviral activity of this drug is the induction of mutation into the viral RNA leading to fatal errors [228]. Clinical trials demonstrated reduced morbidity and mortality in HFRS when treatment was initiated early after HNTV Jameson exposure [226]. However, later initiation of treatment was less effective. A lack of Ribavirin therapeutic efficacy was reported when treating PUUV-infected patients [227]. Another drug used is Favipiravir, an antiviral drug that selectively inhibits the RdRp of negative strand segmented viruses [229]. The efficacy of Favipiravir was demonstrated against SNV and ANDV in vitro and in vivo [230]. Similar to Ribavirin, the protective effect of this drug was demonstrated only when used at the early stage of infection. Late administration failed to reduce virus load and protect hamsters from lethal ANDV infection.

*Targeting the immune response*: Activation of the kinin–kallikrein system and liberation of bradykinin were shown in endothelial cells infected by HNTV and ANDV [231]. This bradykinin was suggested to contribute to increased vascular permeability in orthohantavirus-infected patients. Inhibition of bradykinin was introduced as a novel approach for treating orthohantavirus infection. Icatibant, a blocker of bradykinin binding to its receptor, was demonstrated effective in some case reports. Antonen et al. have shown the efficacy of icatibant in PUUV infection cases, where a single dose of the drug stabilizes the patient’s condition and complete recovery [232]. Similar to this case study, Laine et al. reported a successful outcome using icatibant [233] in another PUUV infection case.

Corticosteroids are most commonly used for treating orthohantavirus infection for their anti-inflammatory effect [234]. Also, decreased corticosteroids were demonstrated in orthohantavirus-infected rats [235], suggesting the role of these hormones in the pathogenesis of this infection. Data from HNTV and PUUV-infected patients supported the hypothesis of corticosteroids’ contribution to the pathogenesis of HFRS. Damage to hypophysis and the adrenal gland could lower cortisol levels and potentially contribute to inflammation [236,237]. This data supported the inclusion of corticosteroids in the HFRS treatment protocol. Rapid recovery was demonstrated in two severe PUUV cases after administering corticosteroids [238]. Also, thrombocyte counts were restored in NE patients after prednisolone treatment was initiated [239,240]. However, the lack of corticosteroid therapeutic effect in ANDV-infected patients was reported by Vial et al. in a double-blind, randomized controlled clinical trial [241].

*Supportive therapy*: Supportive therapy is fundamental for HFRS management. Fluid and electrolyte replacement therapy maintains blood pressure [242]. Thrombocyte transfusion is used in severe thrombocytopenia; however, caution should be exercised to prevent thrombosis [243]. Additionally, continuous renal replacement therapy was effective in patients with multiple organ dysfunction syndromes [244].

## 7. Challenges of Orthohantavirus Diagnostics and Treatment

Orthohantavirus diagnosis is based on clinical presentation, epidemiological data, and detection of serum antibodies. The diagnosis in endemic areas is often based on clinical symptoms and the presence of IgM. However, the expertise of a healthcare provider is essential for early diagnosis and appropriate treatment. Kim and Han reported that 54% of HFRS patients were misdiagnosed at admission [245] due to unusual symptoms. These led to more extended hospitalization than patients with HFRS diagnosed early after admission. HFRS could be misdiagnosed as an acute abdomen [246,247]. It was suggested that this diagnosis could lead to unnecessary surgery and potentially life-threatening complications. In another study, an analysis of 1250 HFRS cases revealed that 13.2% were diagnosed with acute abdomen [248]. Authors suggest that fibro gastroduodenoscopy and diagnostic laparoscopy are optimal for differential diagnosis.

Another challenge could be HFRS diagnosis in children. This is mainly because the disease is commonly mild at this age [249]. Zhang et al. demonstrated two cases of HFRS in children with atypical symptoms [250]. The initial differential diagnosis of systemic lupus erythematosus was made in these patients. Also, differential diagnosis with leptospirosis, rickettsiosis, and heart failure is suggested [251,252]. Leptospirosis is one of the most considered differential diagnoses with HFRS. This is explained by the pathology of leptospirosis, where tubule-interstitial nephritis and thrombocytopenia are common [253,254]. Damage to kidney tissue and decreased thrombocyte counts are also commonly found in HFRS, making clinical symptoms of these diseases reasonably similar. That could be the reason for the misdiagnosis of HFRS, especially in low HFRS endemic regions.

## 8. Conclusions

Orthohantavirus infection is an endemic zoonosis in Asia. Several serotypes, such as HNTV, PUUV, and SEOV, were identified as causing HFRS in China, South Korea, and Japan. China remains the most active HFRS epidemic region, where 90% of all orthohantavirus infections diagnosed worldwide are documented. HFRS clinically is characterized by AKI and increased vascular permeability. There are limited specific treatment options. Therefore, the most effective approach for HFRS management is to prevent infection. Recently, novel orthohantavirus serotypes have been isolated from small animals habituating in India and Thailand. These data provide evidence for the emergence of new genotypes of orthohantaviruses in Asia.

HFRS management remains challenging due to limited therapeutic options specifically targeting orthohantavirus entry and replication. Therefore, the treatment protocol is mainly symptomatic. In this view, the measures to prevent orthohantavirus infection appear essential to control outbreaks. Currently, the only vaccine approved for the prevention of orthohantavirus infection is Hantavax, which could protect against HNTV and SEOV, the orthohantaviruses endemic in Asia. These vaccines could induce long-term immune response with antibodies detected 33 months post-immunization.

Among the novel approaches in treating orthohantavirus infection is using monoclonal antibodies with neutralizing activity against HNTV. These antibodies demonstrated therapeutic efficacy in the I and II phases of clinical studies in the early stage of HFRS. Another novel therapeutic approach is targeting orthohantavirus via αvβ3 integrin receptors.

The emergence of orthohantavirus infection could impact the healthcare system in many Asian countries that have limited experience in diagnosing and treating emerging infections. Therefore, awareness of the circulation of orthohantaviruses and associated healthcare burdens is essential for developing prevention measures. One approach could be integrating screening small mammals for orthohantavirus antibodies and antigens using ELISA or PCR methods into epidemiological surveys. Also, serological testing of febrile patients for anti-orthohantavirus IgM could facilitate early diagnosis of orthohantavirus infection in non-endemic countries. Additionally, analysis of the seroprevalence in the human population could provide a better understanding of orthohantavirus infection prevalence, especially in non-endemic regions.

## Figures and Tables

**Table 1:** Geographic distribution of orthohantaviruses and their host reservoirs.

Orthohantavirus Species	Natural Host Reservoir (Common Name)	Geographic Distribution	Associated Disease	References
Hantaan orthohantavirus (HTNV) *	*Apodemus agrarius* (striped field mouse)	China, Korea, Russia	HFRS	[34]
Dabieshan orthohantavirus (DBSV)	*Niviventer confucianus* (Chinese white-bellied rat)	China	Unknown	[35]
Fugong orthohantavirus (FUGV)	*Eothenomys eleusis* (small oriental vole)	China	Unknown	[36]
Fusong orthohantavirus (FUSV)	*Microtus fortis* (reed vole)	China	Unknown	[37]
Imjin orthohantavirus (MJNV)	*Crocidura lasiura* (Ussuri white-toothed shrew)	China	Unknown	[38,39]
Soochong orthohantavirus (SOOV) *	*Apodemus peninsulae* (Korean field mouse)	Korea	Unknown	[40]
Amur orthohantavirus (AMRV) *	*Apodemus peninsulae* (Korean field mouse)	Asia	HFRS	[40]
Seoul orthohantavirus (SEOV)	*Rattus norvegicus* *Rattus rattus* (rats)	Worldwide	HFRS	[34]
Serang orthohantavirus (SERV)	*Rattus tanezumi* (Asian house rat)	Indonesia	Mild HFRS	[41]
Thailand orthohantavirus (THAIV)	*Bandicoot indica* (Bandicoot rat)	Southeast Asia (Thailand)	HFRS	[42]
Puumala orthohantavirus (PUUV) **	*Myodes glareolus* (Bank vole)	Eurasian continent	Mild HFRS/NE	[43]
Khabarovsk orthohantavirus (KHAV)	*Microtus maximowiczii* (Maximowicz’s vole)	Mongolia, northeast China	unknown	[37]
Hokkaido virus (HOKV) **	*Myodes rufocanus* (Grey red-backed vole)	Japan	HFRS	[44]
Muju virus (MUJI) **	*Myodes regulus* (Royal vole)	South Korea	unknown	[45]
Luxi orthohantavirus (LUXV)	*Eothenomys miletus* (Yunnan red-backed vole)	Asia (China)	unknown	[46]
Jeju orthohantavirus (JJUV)	*Crocidura shantungensis* (Asian lesser white-toothed shrew)	Asia (South Korea)	unknown	[39]
Cao Bang orthohantavirus (CBNV)	*Anourosorex squamipes* (Chinese mole shrew)	China and Vietnam	unknown	[47]
Yakeshi orthohantavirus (YKSV)	*Sorex isodon* (Taiga shrew)	Asia (China)	unknown	[48]
Asama orthohantavirus (ASAV)	*Ulotrichous talpoides* (Japanese shrew mole)	Asia (Japan)	unknown	[49]
Kenkeme orthohantavirus (KKMV)	*Sorex roboratus* (flat-skulled shrew)	Altai (Russia), northeast China	unknown	[50]

* According to ICTV taxonomy, SOOV and AMRV are genetic variants of HNTV. ** According to ICTV taxonomy, HOKV and MUJV are genetic variants of PUUV.

## Data Availability

Not applicable.
